# Two distinct degenerative types of nigrostriatal dopaminergic neuron in the early stage of parkinsonian disorders

**DOI:** 10.1016/j.prdoa.2024.100242

**Published:** 2024-02-15

**Authors:** Tomoya Kawazoe, Keizo Sugaya, Yasuhiro Nakata, Masato Okitsu, Kazushi Takahashi

**Affiliations:** a Department of Neurology, Tokyo Metropolitan Neurological Hospital (TMNH), Tokyo, Japan; b Department of Neuroradiology, TMNH, Tokyo, Japan

**Keywords:** Nigrostriatal dopaminergic degeneration, Neuromelanin-sensitive MRI, Dopamine transporter SPECT, Corticobasal degeneration, Progressive supranuclear palsy, Multiple system atrophy

## Abstract

**Introduction:**

The present study characterized the degeneration of nigrostriatal dopaminergic neurons in the early stages of parkinsonian disorders using integrative neuroimaging analysis with neuromelanin-sensitive MRI and ^123^I-FP-CIT dopamine transporter (DAT) SPECT.

**Methods:**

Thirty-one, 30, and 29 patients with progressive supranuclear palsy (PSP), corticobasal syndrome (CBS) with abnormal specific binding ratio (SBR) in either hemisphere (mean ± 2SD), and parkinsonism-predominant multiple system atrophy (MSA-P), respectively, were enrolled. Neuromelanin-related contrast (NRC) in the substantia nigra (NRC_SN_) and locus coeruleus (NRC_LC_) and the SBR of DAT SPECT were measured. All the patients underwent both examinations simultaneously within five years after symptom onset. After adjusting for interhemispheric asymmetry on neuromelanin-related MRI contrast using the Z-score, linear regression analysis of the NRC_SN_ and SBR was performed for the most- and least-affected hemispheres, as defined by the interhemispheric differences per variable (SBR, NRC_SN_, standardized [SBR + NRC_SN_]) in each patient.

**Results:**

Although the variables did not differ significantly between PSP and CBS, a significant correlation was found for CBS in the most-affected hemisphere for all the definitions, including the clinically defined, most-affected hemisphere. No significant correlation was found between the NRC_SN_ and SBR for any of the definitions in either PSP or MSA-P.

**Conclusion:**

Together with the findings of our previous study of dementia with Lewy bodies (DLB) and Parkinson’s disease (PD), the present findings indicated that neural degeneration in the disorders examined may be categorized by the significance of the NRC_SN_-SBR correlation in PD and CBS and its non-significance in DLB, PSP, and MSA-P.

## Highlights

• Integrative neuroimaging analysis characterized nigrostriatal neuron degeneration.
• Correlation of NRC_SN_ with SBR clearly differed from that of PSP with CBS.
• In MSA-P, interhemispheric laterality of SBR coincided with putamen atrophy.
• No significant correlation occurred between NRC_SN_ and SBR in MSA-P.
• Parkinsonian disorders are divisible into two groups based on NRC_SN_-SBR correlation.

## 1 Introduction

^123^I-FP-CIT dopamine transporter (DAT) single-photon emission computed tomography (SPECT) accurately detects presynaptic dopaminergic deficits and is highly sensitive for the diagnosis of Parkinson’s disease (PD) and parkinsonian syndromes, including dementia with Lewy bodies (DLB), progressive supranuclear palsy (PSP), corticobasal degeneration syndrome (CBS), and parkinsonism-predominant multiple system atrophy (MSA-P) [1]. DAT SPECT had higher sensitivity than ^18^F-FDOPA positron emission tomography (PET) in detecting abnormalities in the early stages of neurodegenerative parkinsonian disorders [2]. Moreover, the widespread application of FDOPA/PET in clinical assessment has been limited by the availability of PET instruments and the radiochemical demands of FDOPA synthesis. DAT imaging may also have a prognostic value for disease progression [3]. However, it has limited value in differentiating the degenerative causes of parkinsonism [1], [4].

Neuromelanin is an insoluble compound that accumulates in large quantities in the catecholamine neurons of the substantia nigra (SN) and locus coeruleus (LC). The neuromelanin content reflects the loss of pigmented neurons in the SN and LC, which are the main pathological hallmarks of PD. Neuromelanin binds to metals, such as iron and copper, and is highly paramagnetic, leading to T1-shortening and hyperintense signaling on T1-weighted turbo spin-echo magnetic resonance imaging (MRI) [5]. Several studies evaluating depigmentation in the SN and LC have recently demonstrated the high sensitivity and specificity of 3-Tesla neuromelanin-sensitive MRI for distinguishing between patients with PD and healthy individuals, providing a potential *in vivo* index of neuromelanin content, disease progression, and neuronal cell loss in the SN and LC [5], [6], [7].

The present study therefore used 3-Tesla neuromelanin-sensitive MRI with ^123^I-FP-CIT DAT SPECT to develop an integrative, neuroimaging analysis for nigrostriatal dopaminergic neuron degeneration. A previous study assessed for any correlation between neuromelanin content in the SN and DAT density in the striatum in the early to intermediate stages of DLB and PD [8]. As a result, the integrative neuroimaging analysis of early-stage PSP, CBS, and MSA-P demonstrated that the five, major, degenerative diseases causing parkinsonism can be divided into two, distinct modes of nigrostriatal dopaminergic neuron degeneration.

## 2 Methods

### 2.1 Participants

Patients with PSP (n = 31), CBS (n = 30) or MSA-P (n = 29) newly diagnosed between January 2015 and December 2021 were retrospectively enrolled.

The inclusion criteria were:

(1) fulfillment of the clinical diagnostic criteria of the Movement Disorder Society for clinically probable PSP [9], the consensus criteria for clinically probable or possible corticobasal degeneration (CBD) [10] or the diagnostic criteria of the Movement Disorder Society for Clinically Established MSA-P [11];
(2) corroboration of the clinical diagnosis by MRI findings assessed by at least two neuroradiologists;
(3) no more than a two-month interval between 3-Tesla neuromelanin-sensitive MRI and ^123^I-FP-CIT DAT SPECT for simultaneous, *in vivo* evaluation;
(4) radiological evaluations conducted within five years after symptom onset to assess the early stages of the target diseases

The exclusion criteria were:

(1) presence of a medical condition (patients with multiple infarcts on MRI which might cause vascular parkinsonism or vascular CBS [12]) or artifact affecting radiological analysis were excluded (e.g., infarct in the basal ganglia); and.
(2) history of use of drugs affecting ^123^I-ioflupane uptake on DAT SPECT.

Although patients with CBS with possible CBD were enrolled, those aged < 50 years at disease onset and those with a family history of any of the diseases were excluded. These criteria were required to diagnose clinically probable CBD [10]. To minimize the potential involvement of Alzheimer’s disease (AD), patients with CBS showing a normal range of the specific binding ratio (SBR) in both hemispheres (mean ± 2SD) (normal DAT SPECT) as compared with that of control subjects in the database [13] were excluded.

Among the control subjects, 19 with essential tremor (ET) satisfying the criteria for definite ET [14] and all the above conditions except for (1) and (4) were enrolled.

The ethics committee of Tokyo Metropolitan Neurological Hospital supervised and approved all the procedures (TS-R03-056), including the use of patient datasets, in accordance with the Declaration of Helsinki. Written informed consent for participation was not required for this retrospective study in accordance with national laws and institutional requirements.

### 2.2 Clinical data and laterality of parkinsonism

All the patients were examined neurologically by at least two neurologists. Clinical data were extracted from medical records, and the dominant side of the parkinsonism was determined based on neurological examination findings.

### 2.3 Integrative neuroimaging analysis for nigrostriatal dopaminergic neuron degeneration

DAT SPECT and 3-Tesla neuromelanin-sensitive MRI were used together less than two months apart as *in vivo,* integrative, neuroimaging analyses of nigrostriatal dopaminergic neuron degeneration. Neuromelanin-related contrast (NRC), SN (NRC_SN_), and LC (NRC_LC_) values and the SBR of DAT SPECT were used as indices of neuromelanin content in the SN and LC and DAT density in the striatum, respectively. The focus was placed on the laterality of the three variables and the relationship between the SBR and NRC_SN_ in each disease.

### 2.4 MRI acquisition

Neuromelanin-sensitive MRI was performed as previously described [15]. All neuromelanin MRI images were obtained using a 3-Tesla MRI system (Discovery MR750; GE Healthcare, Milwaukee, WI, USA). Following the initial localization settings, high-resolution T1-weighted images of the SN and LC were acquired with a two-dimensional fast spin-echo sequence following the procedure in a previous study [8]. The slices were oriented perpendicularly to the brain stem and tilted 20° to the transaxial plane through the anterior and posterior commissures to minimize the partial volume effect on tissues other than the SN [16].

### 2.5 Postprocessing of the neuromelanin MRI data

The analyses measured the NRC_SN_, defined as the number of pixels of high-signal intensity areas on T1-weighted imaging of the SN. In brief, as previously described [8], an attempt was made to remove background noise. The three, contiguous, axial slices of the SN were converted to eight-bit gray scale images using Image J software (version 1.52, NIH, Bethesda, Maryland, USA). An optimized threshold was semiautomatically determined by a blinded investigator as previously described [8]. After setting the optimized threshold, the pixel numbers in the SN were automatically calculated, yielding the NRC_SN_ value.

Similarly, NRC_LC_ was measured with ImageJ software using the number of pixels in the high signal intensity areas on T1-weighted imaging of the LC. After converting the MRI data into an eight-bit file, images containing the pons were smoothed to identify areas of high-intensity signaling in the LC. The region of interest was then located in the pons tegmentum to derive the individual background noise. An optimized threshold was semiautomatically determined when the noise was equal to zero in a manner similar to the measurement of the NRC_SN_. The number of pixels in high signal intensity areas on the right and left sides of the LC (NRC_LC_) was measured in two, contiguous, axial slices.

### 2.6 DAT SPECT

DAT SPECT was performed as previously described [17]. Briefly, SPECT images were reconstructed using an iterative algorithm on DAT SPECT images. The reconstructed data were quantified using DaTView (Nihon Medi-Physics, Tokyo, Japan) and the Southampton method [18]. The SBR was calculated as follows:

SBR=(striatumuptake-wholebrainuptake)/(wholebrainuptake)

In this method, geometrical volumes of interest larger than the striatum are used to consider partial volume effects [17]. Radiological technicians were not allowed to define the striatum manually, and their interventions were limited to shifting the template geometrical volumes of interest. The Southampton method is highly reproducible, with an operator-introduced variability of only 4 % [18]. The original SBR was calibrated as previously described [16].

### 2.7 Statistical analysis

Continuous variables were assessed for normality and homogeneity of variance using the Shapiro-Wilk and Levene’s tests. Normally distributed data were analyzed using Student’s *t*-test or Welch’s *t*-test. The Mann-Whitney *U* test was used for non-normally distributed variables. Significance was set at *P* < 0.05. Contingency analysis was performed using Fisher’s exact test. For multiple comparisons, the Games-Howell test was used after one-way analysis of variance (parametric) to correct for the false discovery rate (=0.05). The Steel-Dwass test was used after the Kruskal-Wallis test (non-parametric) as a post-hoc test.

The residuals of linear regression analysis were assessed for normality, linearity, and homoscedasticity using residual diagnostic plots after fitting the linear regression analysis and Shapiro-Wilk test. For multiple comparisons of the linear regression analyses, Bonferroni correction was performed to correct the false discovery rate (=0.05). All statistical analyses were performed using IBM SPSS Statistics for Windows, version 20 (IBM Corp., Armonk, N.Y., USA).

## 3 Results

### 3.1 Clinical characteristics of patients with PSP and those with CBS

Table 1 summarizes the demographic characteristics of the PSP and CBS groups. The mean disease duration of PSP and CBS at the radiological evaluations with neuromelanin-sensitive MRI and DAT SPECT was 2.3 ± 1.3 and 2.5 ± 1.2 years (mean ± SD), respectively. There was no significant difference in the sex ratio or age at MRI/SPECT among the PSP, CBS, and control groups. The clinical phenotypes of PSP were PSP-Richardson's syndrome (PSP-RS, n = 22), PSP-parkinsonism (n = 6), PSP with progressive gait freezing (n = 2), and PSP with predominant speech/language disorder (n = 1). According to diagnostic criteria published by Armstrong et al. [10], the clinical phenotypes of CBS were CBS (probable CBS, n = 4; possible CBS, n = 15), PSP syndrome (n = 10), and non-fluent/agrammatic variants of primary progressive aphasia (n = 1).

**Table 1:** Demographic features and imaging findings in the control, PSP, and CBS groups.

	PSP	CBS	ET (Control)	*P^a^* value PSP vs CBS
Number	31	30	19	
M:F ratio	18: 13 *P* = 0.063	13: 17 *P* = 0.303	6: 13−	0.186
Age at image acquisition	77.3 ± 5.7 *P* = 0.416	73.2 ± 8.1 *P* = 0.631	74.8 ± 7.0 −	0.061
Disease duration at MRI (years)	2.4 ± 1.3	2.5 ± 1.2		0.629
Disease duration at SPECT (years)	2.4 ± 1.3	2.5 ± 1.2		0.697

Imaging findings				
SBR Right	1.78 ± 0.89 *P* = 0.000*	2.17 ± 1.02 *P* = 0.000*	3.90 ± 0.83−	0.251
Left	1.76 ± 0.87 *P* = 0.000*	1.93 ± 1.06 *P* = 0.000*	3.84 ± 0.75−	0.782
NRC_SN_ Right	12.71 ± 6.56 *P* = 0.000*	15.26 ± 9.50 *P* = 0.000*	31.01 ± 9.12−	0.703
Left	17.01 ± 6.81 *P* = 0.000*	22.59 ± 15.89 *P* = 0.000*	42.31 ± 9.41−	0.742
NRC_LC_ Right	2.17 ± 1.09 *P* = 0.974	1.91 ± 1.18 *P* = 0.632	2.25 ± 1.31−	0.643
Left	2.72 ± 1.36 *P* = 0.499	2.62 ± 1.01 *P* = 0.280	3.14 ± 1.20−	0.946

Data on continuous variables are expressed as the mean ± SD. *P^a^*: *P* value for comparison between the PSP and CBS groups. The *P* value represents the result of a comparison between the control subjects and the PSP and CBS groups. * *P* < 0.05 indicates statistical significance. Abbreviations: CBS, corticobasal syndrome; ES, essential tremor; NRC_LC_, neuromelanin-related contrast in the locus coeruleus; NRC_SN_, neuromelanin-related contrast in the substantia nigra; PSP, progressive supranuclear palsy; SBR, specific binding ratio.

### 3.2 SBR, NRC_SN_, and NRC_LC_, in the PSP and CBS groups

**Fig. 1:**
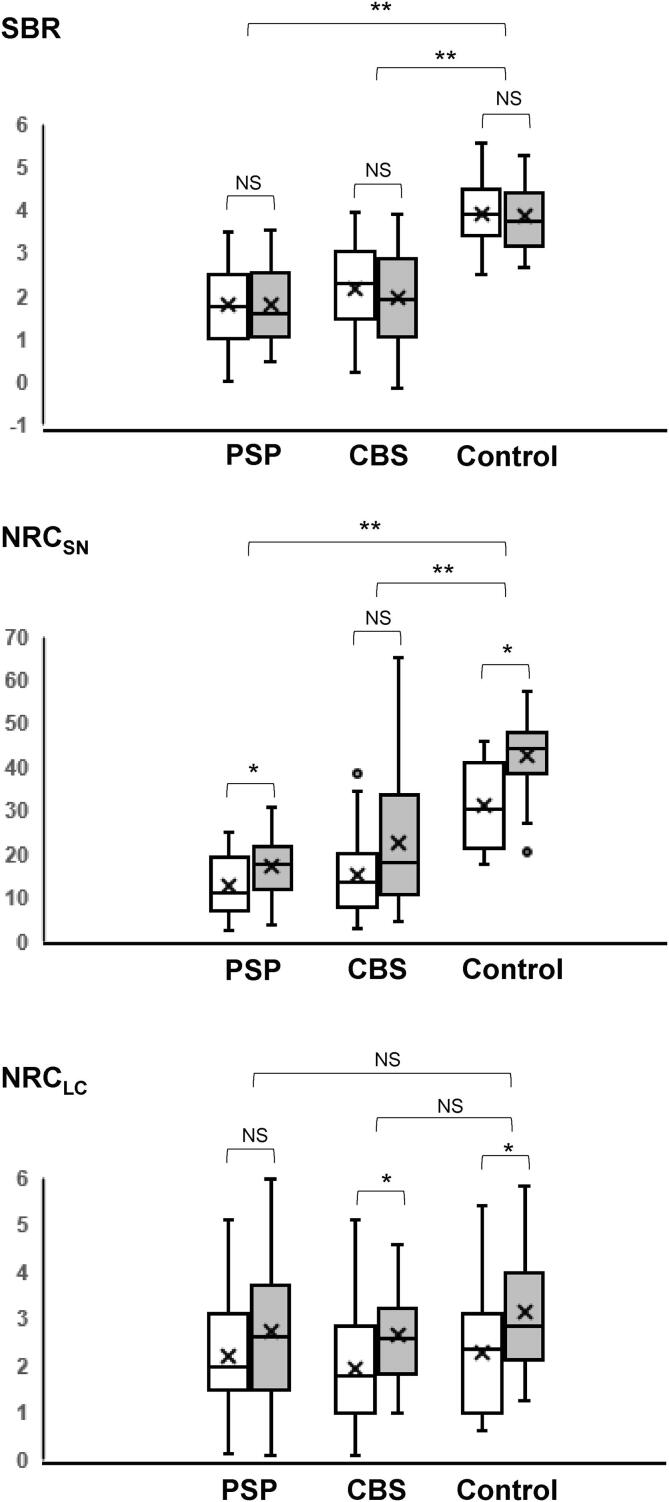
Box plots showing SBR, NRC_SN_, and NRC_LC_ distribution. Box plots showing the distribution of SBR, NRC_SN_, and NRC_LC_ in the right (open) and left (gray) hemispheres of patients with PSP, CBS, and control subjects. The line in the box represents the median value, and x represents the mean value. * p < 0.05, ** p < 0.01. Abbreviations: SBR, specific binding ratio; NRC_SN_, neuromelanin-related contrast in the substantia nigra; NRC_LC_, neuromelanin-related contrast in the locus coeruleus; PSP, progressive supranuclear palsy; CBS, corticobasal syndrome; NS, non-significant.

Compared with the control group, the PSP and CBS groups demonstrated a significant bilateral reduction in the SBR and NRC_SN_ whereas the difference in the NRC_LC_ was non-significant (Table 1). There was also no significant difference in the SBR, NRC_SN_ or NRC_LC_ between the PSP and CBS groups in either hemisphere. A significant difference was found in the interhemispheric values of the NRC_SN_ and NRC_LC_ in all three groups except for NRC_SN_ in CBS (*P* = 0.063) and NRC_LC_ in PSP (*P* = 0.083) whereas no significant difference was found in the SBR between the hemispheres (Fig. 1).

### 3.3 Correction of the right-left asymmetry of neuromelanin-sensitive MRI

A significant difference was found in the NRC in the SN and/or LC between the right and left hemispheres not only in the disease groups but also in the control subjects. The interhemispheric asymmetry of the NRC in the SN and LC might affect the correlation between the NRC_SN_ and the SBR. Therefore, the Z-score was used to correct the interhemispheric asymmetry of the NRC as described in a previous study [8]. Using linear regression to assess the relationship between NRC_LC_ and the natural, logarithmic transformation of NRC_SN_ (In(NRC_SN_) in the control group, each value of the predicted In(NRC_SN_) was set to zero (the mean). Then, the Z-score of each actual In(NRC_SN_) value was calculated in terms of the standard deviation from the mean of the corresponding residuals.

### 3.4 Linear regression analysis of the corrected NRC_SN_ and SBR values of the most-affected and least-affected sides in the PSP and CBS groups

**Fig. 2:**
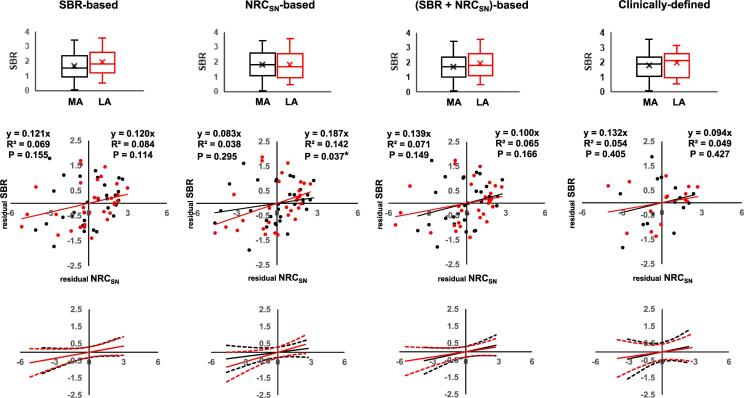
Linear regression analysis of the corrected NRC_SN_ and SBR in the most and least affected (MA/LA) sides in PSP. Using the Z-score of In(NRC_SN_), linear regression analysis was performed for the corrected NRC_SN_ and SBR on the SBR-based MA (black) and LA (red) sides, the NRC_SN_-based MA and LA sides, the (SBR + NRC_SN_)-based MA and LA sides, and clinically defined MA and LA sides. Linear regression for the clinically defined MA and LA sides was performed in the subtype of PSP: PSP-RS with laterality of parkinsonism (left-dominant parkinsonism, n = 5; right-dominant parkinsonism, n = 10). Upper portion: Box plots of the distribution of SBR showing a comparison of the values. The line in the box represents the median value, and x represents the mean value. Middle portion: Results of linear regression analysis of the corrected NRC_SN_ (residual NRC_SN_) and SBR (residual SBR) values after adjusting for individual differences in age. Lower portion: 95 % confidence interval for each linear model (broken line). Abbreviations: MA, most affected; LA, least affected; SBR, specific binding ratio; NRC_SN_, neuromelanin-related contrast in the substantia nigra; PSP, progressive supranuclear palsy; PSP-RS, PSP-Richardson's syndrome. (For interpretation of the references to colour in this figure legend, the reader is referred to the web version of this article.)

**Fig. 3:**
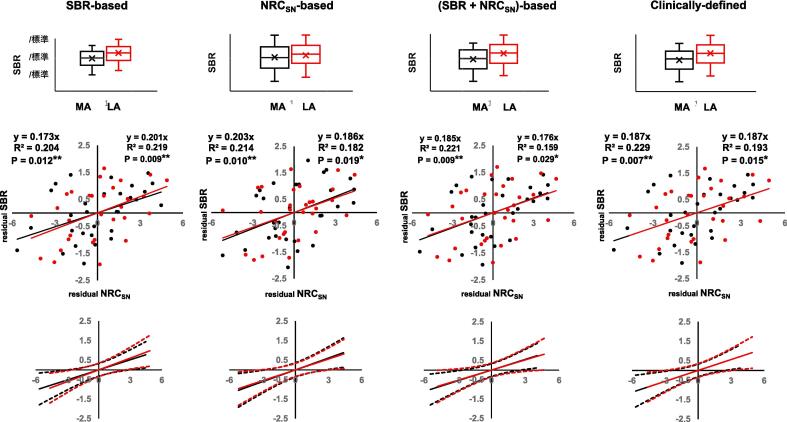
Linear regression analysis of the corrected NRC_SN_ and SBR in the most and least affected (MA and LA) sides in CBS. Linear regression analysis of the corrected NRC_SN_ and SBR in the SBR-based MA (black) and LA (red) sides, NRC_SN_-based MA and LA sides, (SBR + NRC_SN_)-based MA and LA sides, and clinically defined, MA and LA sides (left-dominant parkinsonism, n = 13; right-dominant parkinsonism, n = 17) was performed using the Z-score of the In(NRC_SN_). Upper portion: Box plots of the distribution of SBR showing a comparison of the values. The line in the box represents the median value, and x represents the mean value. Middle portion: Results of linear regression analysis of the corrected NRC_SN_ (residual NRC_SN_) and SBR (residual SBR) after adjusting for individual differences in age. Lower portion: 95 % confidence interval for each linear model (broken line). Abbreviations: MA, most affected; LA, least affected; SBR, specific binding ratio; NRC_SN_, neuromelanin-related contrast in the substantia nigra; CBS, corticobasal syndrome. ** Significant result following Bonferroni false discovery rate correction. (For interpretation of the references to colour in this figure legend, the reader is referred to the web version of this article.)

Fig. 2, Fig. 3 show the results of linear regression analysis assessing the relationship between the corrected NRC_SN_ (residual NRC_SN_) and SBR (residual SBR) in the PSP and CBS group, respectively, after removing the effects of age on the SBR and the NRC_SN_. To compare the correlation between the PSP and CBS groups, the SBR of the hemispheres in each participant was used to define the SBR-based, most affected and least affected sides (most affected side: low SBR; least affected side: high SBR) (Fig. 2, Fig. 3, left column). The NRC_SN_ between the hemispheres was used to define the NRC_SN_-based, most affected and least affected sides (Fig. 2, Fig. 3, middle left column). The (SBR + NRC_SN_)-based, most affected and least affected sides were defined using a combination of values (standardized SBR + standardized NRC_SN_) of each hemisphere (Fig. 2, Fig. 3, middle right column). The clinically defined, most affected and least affected sides were also used (Fig. 2, Fig. 3, right column). Nearly half the patients with PSP had no apparent laterality of parkinsonism while all the patients with CBS did. The clinical phenotypes of PSP patients with laterality of parkinsonism (n = 17) were PSP-RS (n = 15) and PSP-parkinsonism (n = 2). Therefore, linear regression based on the laterality of parkinsonism (clinically defined, most affected and least affected sides) was performed in the subtype of PSP: PSP-RS with laterality of parkinsonism (Fig. 2, right column, n = 15).

Among the most affected sides in PSP, the highest correlation coefficient of the NRC_SN_ with the SBR was observed on the (SBR + NRC_SN_)-based, most affected side (*R*^2^ = 0.071, *P* = 0.149) although it did not attain statistical significance. In CBS, the NRC_SN_ significantly correlated with the SBR in all the most affected sides. Among these, the highest correlation was observed in the clinically-defined, most affected side (*R*^2^ = 0.229, *P* = 0.007).

Furthermore, linear regression was performed between the corrected NRC_SN_ and SBR in the clinical subtypes of the PSP group (PSP-RS, n = 22) and CBS group (CBS, n = 19; PSP syndrome, n = 10). For the most affected side in PSP-RS, the highest correlation coefficient of the NRC_SN_ with the SBR was observed on the SBR-based, most affected side (*R*^2^ = 0.157, *P* = 0.068) (Supplemental Table 1, Supplemental Fig. 1). In CBS and PSP syndrome, the highest correlation coefficient was observed on the NRC_SN_-based, most affected side (*R*^2^ = 0.247, *P* = 0.030) and on the clinically defined, most affected side (*R*^2^ = 0.552, *P* = 0.014), respectively (Supplemental Table 1, Supplemental Fig. 1). Although the correlations did not attain statistical significance after Bonferroni correction for multiple comparisons due to the small number of patients, these results suggested that PSP-RS and PSP syndrome, the clinical subtypes of PSP and CBS, largely contributed to the difference in the correlation of the NRC_SN_ with the SBR between the PSP and CBS groups.

### 3.5 Clinical characteristics and imaging findings of the MSA-P group

Supplemental Table 2 summarizes the demographic features and imaging findings of the MSA-P and age-matched control groups. The mean disease duration at the radiological evaluations was 2.7 ± 1.5 years in the MSA-P group. Compared with the control group, a significant reduction in the NRC_SN_ and NRC_LC_ was observed in the MSA-P group. A significant difference was also found in the NRC_SN_ (*P* = 0.044) and NRC_LC_ (*P* = 0.002) between the right and left hemispheres in both groups whereas no significant difference in the SBR was found between the hemispheres.

A highly sensitive method (coronal fluid attenuated inversion-recovery image on 3-Tesla MRI) for detecting atrophy in the posterior putamen [19] found atrophy in the posterior putamen of 25 patients with MSA-P (25/29) on the radiological evaluations using neuromelanin-sensitive MRI and DAT SPECT. Atrophy developed in the putamen of four patients although an initial examination for atrophy returned negative. The hemispheric laterality of atrophy in the putamen in each patient was markedly consistent with that of the SBR values (23/23). The remaining, two patients demonstrated no clear, hemispheric difference in the degree of atrophy of the posterior putamen.

### 3.6 Linear regression analysis of the corrected NRC_SN_ and SBR of the most-affected and least-affected sides in the MSA-P group

Similarly, various definitions were established based on interhemispheric differences in each variable. Supplemental Fig. 2 shows the results of linear regression assessing the relationship between the corrected NRC_SN_ (residual NRC_SN_) and SBR (residual SBR) in 29 patients with MSA-P after removing the effects of age. The highest correlation between the NRC_SN_ and SBR was observed on the NRC_SN_-based, most affected side although it did not reach statistical significance (*P* = 0.082).

Supplemental Table 3 summarizes the results of linear regression analysis of the corrected NRC_SN_ and SBR in PSP, CBS, and MSA-P, along with the results from 29 patients with DLB and 52 patients with PD derived from a previous study [8], which together cover the five major degenerative diseases causing parkinsonism.

## 4 Discussion

The clinical diagnosis of CBS is challenging. Various, neurodegenerative disorders may be present in patients diagnosed with CBS. In a previous study of the background pathologies of CBS, CBD (38%) was followed in frequency by two, major diseases, PSP (24%) and AD (15%) [20]. Other, neuropathological findings in patients with CBS were Lewy body disease, frontotemporal lobar degeneration with TDP-43 inclusions, motor neuron disease, frontotemporal lobar degeneration with fused-in-sarcoma pathology, cerebrovascular disease, Creutzfeldt-Jakob disease**,** and atypical MSA [20], [21]. Thus, CBS is a group of pathologies linked by the same clinical manifestation. The exact mechanism leading to a concurrent syndrome despite multiple pathologies remains unclear. A recent study reported dyslipidemia in CBS, even in patients without significant vascular changes in MRI, suggesting a possible role of vascular pathology in CBS development [22]. It is still unclear which clinical features can predict the underlying pathology in CBS. Using AD-related biomarkers, Litvan et al. pointed out that the specificity of the CBD criteria proposed by Armstrong could be improved [23]. However, differentiating non-AD tauopathies, especially PSP and CBD, remains a challenge [21].

Recently, several studies examining the utility of DAT SPECT for differentiating between DLB and AD reported no significant reduction in the SBR of DAT SPECT for AD but did find a significant reduction for DLB in comparison with control subjects [24], [25]. Therefore, after excluding the normal range of the SBR in the present CBS cohort, both CBD and PSP were assumed to be major diseases in the background pathology. A clear difference in the correlation between NRC_SN_ and the SBR between the PSP and CBS groups suggested that these two conditions involve different modes of nigrostriatal dopaminergic neuronal degeneration. In a recent, retrospective, multicentric study, Aiba et al. demonstrated the frequency of clinical phenotypes based on Armstrong’s criteria in 32 patients with pathologically, genetically, and biochemically verified CBD [26]. Among the four, clinical subtypes associated with the pathology of CBD, the most common was PSP syndrome followed by frontal behavioral-spatial syndrome and possible CBS. Patients with PSP-RS and PSP syndrome share many clinical features, and no single clinical feature is sufficient to predict the underlying pathology [21], [26]. The results of our linear regression between the NRC_SN_ and SBR in the clinical subtypes of PSP and CBS further suggested that PSP-RS and PSP syndrome might differ in the degenerative process of nigrostriatal dopaminergic neurons (Supplemental Table 1, Supplemental Fig. 1) although a large cohort study would be required to clarify the findings.

In MSA-P, the absence of a significant correlation between NRC_SN_ and the SBR for any of the definitions examined (Supplemental Fig. 2) suggested that the loss of the soma (represented by NRC_SN_) and that of the presynaptic terminals (represented by SBR) in nigrostriatal dopaminergic neurons might occur independently. In contrast, the interhemispheric laterality of atrophy in the posterior putamen in each MSA-P patient was highly consistent with the interhemispheric laterality of the SBR values. Thus, in MSA-P, degeneration of the putamen may cause presynaptic nigrostriatal dopamine dysfunction by *trans*-synaptic retrograde degeneration, which well explains L-DOPA-resistant parkinsonism.

Degeneration of nigrostriatal dopaminergic neurons is the main pathological finding in PD and is also a major factor in DLB, PSP, CBD, and MSA-P. These disorders share many clinical features and show a high degree of variation among individuals, making them difficult to diagnose, especially in their early stages. Taken together with our previous study of DLB and PD [8], in terms of the correlation between the NRC_SN_-SBR, the present study demonstrated that the five, neurodegenerative diseases causing parkinsonism can be divided into two, distinct groups (significant in PD and CBS and non-significant in DLB, PSP, and MSA-P). Both PD and CBS demonstrated a significant correlation between the NRC_SN_ and SBR in all instances of the most affected side (Supplemental Table 3). These diseases are characterized by persistent asymmetry of parkinsonism. Thus, the tightly connected degeneration of the soma (represented by NRC_SN_) and presynaptic terminals (represented by SBR) in both PD and CBS raises the possibility of an association between the asymmetry of parkinsonism and the vulnerability of hyperbranched axons, a unique morphological feature of the nigrostriatal dopaminergic neuron [27], to pathological alpha synuclein / tau aggregation. The SN pars compacta is subdivided into three compartments based on their functional and anatomical organization (sensorimotor, associative, and limbic), all of which have distinct connections to the striosome and matrix compartments of the striatum [28]. Further study of integrative neuroimaging of the subregions of the SN and striatum in parkinsonian diseases will clarify the degenerative patterns of nigrostriatal dopaminergic neurons in each disease and possibly help contribute to distinguishing these disorders in their early stages.

The present study has several limitations:

(1) This study was retrospective and enrolled a relatively small number of patients because neuromelanin-sensitive MRI and DAT SPECT had to be performed simultaneously.
(2) Four patients with CBS with possible CBD showing a normal SBR range in both hemispheres on DAT SPECT were excluded although they might have had CBD.
(3) Previous studies demonstrated that DAT SPECT and neuromelanin-sensitive MRI can distinguish between ET and PD [29], [30]. Dopaminergic and iron imaging did not demonstrate a substantial overlap between the pathophysiology of ET and that of PD [31]. In a previous study, no obvious changes were observed in any brain region, except the cerebellum, in the postmortem findings of ET [32]. Therefore, the present study used data from patients with ET as the control but did not obtain 3-Tesla neuromelanin-sensitive MRI and DAT SPECT for age-matched healthy controls.

## CRediT authorship contribution statement

**Tomoya Kawazoe:** Data curation, Formal analysis, Investigation, Writing – original draft, Writing – review & editing. **Keizo Sugaya:** Conceptualization, Data curation, Formal analysis, Funding acquisition, Investigation, Methodology, Supervision, Writing – original draft, Writing – review & editing. **Yasuhiro Nakata:** Data curation, Formal analysis, Investigation, Writing – review & editing. **Masato Okitsu:** Data curation, Investigation, Writing – review & editing. **Kazushi Takahashi:** Conceptualization, Data curation, Supervision, Writing – review & editing.

## Declaration of competing interest

The authors declare that they have no known competing financial interests or personal relationships that could have appeared to influence the work reported in this paper.

## References

[1] Kägi G., Bhatia K.P., Tolosa E. (2010). The role of DAT-SPECT in movement disorders. J Neurol Neurosurg Psychiatry.

[2] Wallert E., Letort E., van der Zant F., Winogrodzka A., Berendse H., Beudel M. (2022). Comparison of [18F]-FDOPA PET and [123I]-FP-CIT SPECT acquired in clinical practice for assessing nigrostriatal degeneration in patients with a clinically uncertain parkinsonian syndrome. EJNMMI Res..

[3] Trinh I., Muralidhar A., Yang J., Phielipp N. (2023). Quantified striatal dopaminergic denervation as predictor for motor outcomes in parkinson's disease. Mov Disord Clin Pract.

[4] Piccini P., Whone A. (2004). Functional brain imaging in the differential diagnosis of Parkinson's disease. Lancet Neurol.

[5] Sulzer D., Cassidy C., Horga G., Kang U.J., Fahn S., Casella L. (2018). Neuromelanin detection by magnetic resonance imaging (MRI) and its promise as a biomarker for Parkinson's disease. NPJ Parkinsons Dis.

[6] Sasaki M., Shibata E., Tohyama K., Takahashi J., Otsuka K., Tsuchiya K. (2006). Neuromelanin magnetic resonance imaging of locus ceruleus and substantia nigra in Parkinson's disease. Neuroreport.

[7] Ohtsuka C., Sasaki M., Konno K., Koide M., Kato K., Takahashi J., J. (2013). Changes in substantia nigra and locus coeruleus in patients with early-stage Parkinson's disease using neuromelanin-sensitive MR imaging. Neurosci Lett.

[8] Okitsu M., Sugaya K., Nakata Y., Kawazoe T., Ikezawa J., Okiyama R. (2023). Degeneration of nigrostriatal dopaminergic neurons in the early to intermediate stage of dementia with Lewy bodies and Parkinson's disease. J Neurol Sci.

[9] Höglinger G.U., Respondek G., Stamelou M., Kurz C., Josephs K.A., Lang A.E. (2017). Clinical diagnosis of progressive supranuclear palsy: Movement Disorder Society criteria. Mov Disord.

[10] Armstrong M.J., Litvan I., Lang A.E., Bak T.H., Bhatia K.P., Borroni B. (2013). Criteria for the diagnosis of corticobasal degeneration. Neurology.

[11] Wenning G.K., Stankovic I., Vignatelli L., Fanciulli A., Calandra-Buonaura G., Seppi K. (2022). Movement disorder society criteria for the diagnosis of multiple system atrophy. Mov Disord.

[12] Koga S., Roemer S.F., Kasanuki K., Dickson D.W. (2019). Cerebrovascular pathology presenting as corticobasal syndrome: an autopsy case series of “vascular CBS”. Parkinsonism Relat Disord.

[13] Matsuda H., Murata M., Mukai Y., Sako K., Ono H., Toyama H. (2018). Japanese multicenter database of healthy controls for [123I] FP-CIT SPECT. Eur J Nucl Med Mol Imaging.

[14] Hopfner F., Haubenberger D., Galpern W.R., Gwinn K., Van't Veer A., White S. (2016). Knowledge gaps and research recommendations for essential tremor. Parkinsonism Relat Disord.

[15] Nakata Y., Sakamoto A., Kawata A. (2022). Neuromelanin imaging analyses of the substantia nigra in patients with Machado-Joseph disease. Neuroradiology.

[16] Kawaguchi H., Shimada H., Kodaka F., Suzuki M., Shinotoh H., Hirano S. (2016). Principal component analysis of multimodal neuromelanin MRI and dopamine transporter PET data provide a specific metric for nigral dopaminergic neuronal density. PLoS One.

[17] Ikezawa J., Yokochi F., Okiyama R., Kumada S., Tojima M., Kamiyama T. (2021). Is generalized and segmental dystonia accompanied by impairments in the dopaminergic system?. Front Neurol.

[18] Tossici-Bolt L., Hoffmann S.M., Mehta R.L., Fleming J.S. (2006). Quantification of [123I]FP-CIT SPECT brain images: an accurate technique for measuring specific binding ratios. Eur J Nucl Med Mol Imaging.

[19] Mori K., Yagishita A., Shimizu T. (2023). Asymmetrical putaminal atrophy in parkinsonism-predominant multiple system atrophy (MSA-P): a case report. Radiol Case Rep.

[20] Shir D., Thu Pham N.T., Botha H., Koga S., Kouri N., Ali F. (2023). Clinicoradiologic and neuropathologic evaluation of corticobasal syndrome. Neurology.

[21] Koga S., Josephs K.A., Aiba I., Yoshida M., Dickson D.W. (2022). Neuropathology and emerging biomarkers in corticobasal syndrome. J Neurol Neurosurg Psychiatry.

[22] Madetko-Alster N., Alster P., Bartošová T., Klempíř J., Migda B., Przewodowska D. (2023). Could hyperlipidemia be a risk factor for corticobasal syndrome? - a pilot study. Neurol Neurochir Pol.

[23] Litvan I., Lang A.E., Armstrong M. (2022). CBD diagnostic criteria: exclusions as important as inclusions. J Neurol Neurosurg Psychiatry.

[24] Walker Z., Costa D.C., Walker R.W., Shaw K., Gacinovic S., Stevens T. (2002). Differentiation of dementia with Lewy bodies from Alzheimer's disease using a dopaminergic presynaptic ligand. J Neurol Neurosurg Psychiatry.

[25] Shimizu S., Hirao K., Kanetaka H., Namioka N., Hatanaka H., Hirose D. (2016). Utility of the combination of DAT SPECT and MIBG myocardial scintigraphy in differentiating dementia with Lewy bodies from Alzheimer's disease. Eur J Nucl Med Mol Imaging.

[26] Aiba I., Hayashi Y., Shimohata T., Yoshida M., Saito Y., Wakabayashi K. (2023). Clinical course of pathologically confirmed corticobasal degeneration and corticobasal syndrome. Brain Commun.

[27] Matsuda W., Furuta T., Nakamura K.C., Hioki H., Fujiyama F., Arai R. (2009). Single nigrostriatal dopaminergic neurons form widely spread and highly dense axonal arborizations in the neostriatum. J Neurosci.

[28] Zhang Y., Larcher K.M., Misic B., Dagher A. (2017). Anatomical and functional organization of the human substantia nigra and its connections. Elife.

[29] Waln O., Wu Y., Perlman R., Wendt J., Van A.K., Jankovic J. (2015). Dopamine transporter imaging in essential tremor with and without Parkinsonian features. J Neural Transm. (vienna).

[30] Booth T.C., Nathan M., Waldman A.D., Quigley A.M., Schapira A.H., Buscombe J. (2015). Role of functional dopamine-transporter SPECT imaging in parkinsonian syndromes, part 2. AJNR Am J Neuroradiol.

[31] Holtbernd F., Shah N.J. (2021). Imaging the Pathophysiology of Essential Tremor-A Systematic Review. Front Neurol.

[32] Louis E.D., Faust P.L. (2020). Essential tremor pathology: neurodegeneration and reorganization of neuronal connections. Nat Rev Neurol.

