# Supplementary material for: Two distinct degenerative types of nigrostriatal dopaminergic neuron in the early stage of parkinsonian disorders
Source: Clin Park Relat Disord. 2024 Feb 15;10:100242. doi: 10.1016/j.prdoa.2024.100242 (PMC10883825; doi:10.1016/j.prdoa.2024.100242)
Supplement: Supplementary data 1 [file mmc1.docx]

**Supplemental File**

**Supplemental Table 1.**

**Supplemental Figure 1.**

**Supplemental Table 2.**

**Supplemental Table 3.**

**Supplemental Figure 2.**

**Two distinct degenerative types of nigrostriatal dopaminergic neuron in the early stage of parkinsonian disorders**

Tomoya Kawazoe, Keizo Sugaya, Yasuhiro Nakata, Masato Okitsu, Kazushi Takahash^1^

Corresponding author:

Keizo Sugaya

Department of Neurology

Tokyo Metropolitan Neurological Hospital

**Supplemental Table 1. Linear regression between the NRC_SN_ and SBR in the clinical subtypes of PSP and CBS**

|  | PSP | CBS | |
| --- | --- | --- | --- |
|  | RS  n = 22 | CBS  n = 19 | PSP syndrome  n = 10 |
| SBR-based  MA side | *R*^2^ = 0.157  *P* = 0.068 | *R*^2^ = 0.152  *P* = 0.100 | *R*^2^ = 0.513  *P* = 0.020* |
| NRC_SN_-based  MA side | *R*^2^= 0.063  *P* = 0.261 | *R*^2^ = 0.247  *P* = 0.030* | *R*^2^ = 0.344  *P* = 0.075 |
| (SBR + NRC_SN_)-based MA side | *R*^2^= 0.132  *P* = 0.096 | *R*^2^ = 0.159  *P* = 0.091 | *R*^2^ = 0.353  *P* = 0.070 |
| Clinically-defined MA side | - | *R*^2^ = 0.152  *P* = 0.100 | *R*^2^ = 0.552  *P* = 0.014* |

After adjusting for the interhemispheric asymmetry of neuromelanin-related MRI contrast by using the Z-score, age-adjusted linear regression analysis of the NRC_SN_ and SBR was performed for the most affected and least affected sides of the hemispheres for the various definitions. The other clinical subtypes of PSP and CBS were not performed due to the small number of patients. *Significance was set at *P* <0.05. Abbreviations: CBS, corticobasal syndrome; LA, least affected; MA, most affected; NRC_SN_, neuromelanin-related contrast in the substantia nigra; PSP, progressive supranuclear palsy; *R*^2^, coefficient of determination; RS, Richardson's syndrome: SBR, specific binding ratio.


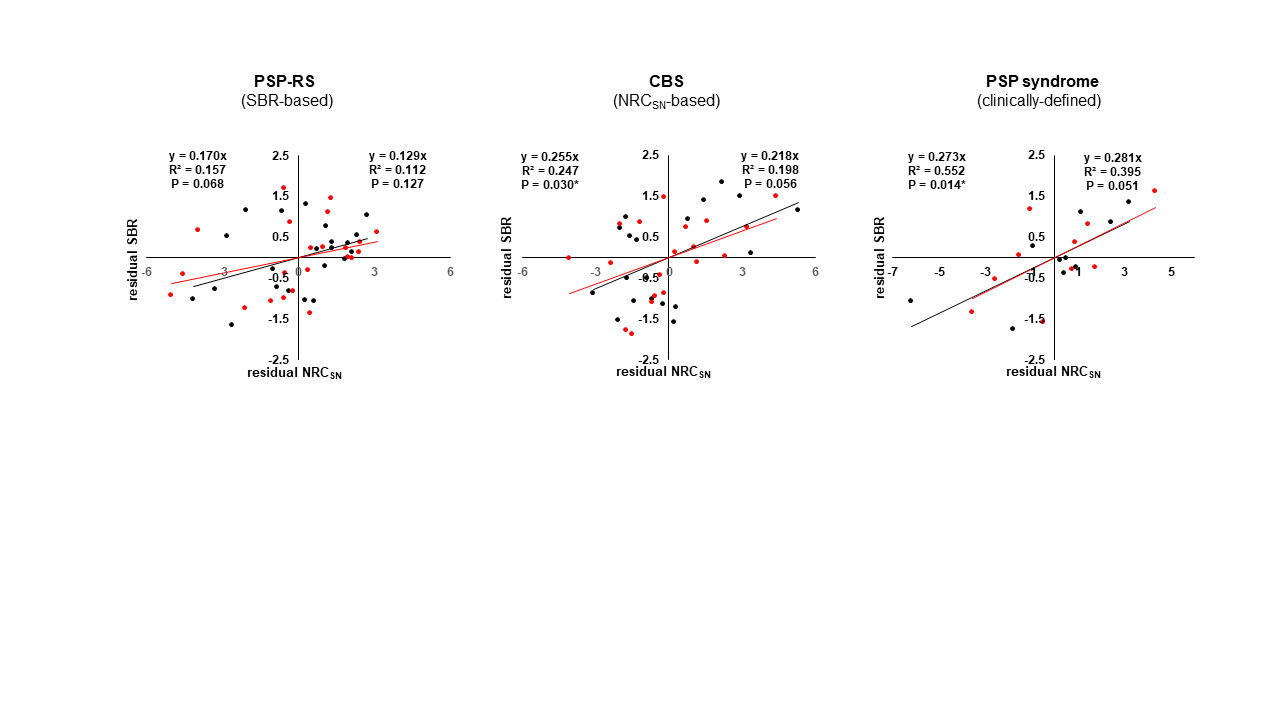


**Supplemental Figure 1.**

**Representative results of linear regression between the corrected NRC_SN_ and SBR in the clinical subtypes of PSP and CBS**

Linear regression between the corrected NRC_SN_ (residual NRC_SN_) and SBR (residual SBR) on the most affected (black) and least affected sides (red) after adjusting for individual differences in age was performed in the clinical subtypes of PSP and CBS groups (PSP-RS, n = 22; CBS, n = 19; PSP syndrome, n = 10). In the PSP-RS, the highest correlation coefficient of NRC_SN_ with SBR was observed on the SBR-based, most affected side (left column, *R*^2^ = 0.157, *P* = 0.068). In CBS and PSP syndrome, the clinical subtypes of CBS, the highest correlation coefficient was observed on the NRC_SN_-based, most affected side (middle column, *R*^2^ = 0.247, *P* = 0.030) and on the clinically defined, most affected side (right column, *R*^2^ = 0.552, *P* = 0.014), respectively. Abbreviations: CBS, corticobasal syndrome; NRC_SN_, neuromelanin-related contrast in the substantia nigra; PSP, progressive supranuclear palsy; PSP-RS, PSP-Richardson's syndrome; *R*^2^, coefficient of determination: SBR, specific binding ratio.

**Supplemental Table 2. Demographic features and imaging findings in the control and MSA-P groups**

|  | **MSA-P** | **Control^2)^** | **P value** |
| --- | --- | --- | --- |
| **Number** | 29 | 22 |  |
| **M:F ratio** | 10 : 19 | 8 : 14 | 0.560 |
| **Age at image** **acquisition** | 68.2 ± 8.3 | 67.4 ± 8.9 | 0.797 |
| **Disease duration at MRI (years)** | 2.7 ± 1.5 | - | - |
| **Disease duration at SPECT (years)** | 2.7 ± 1.5 | - | - |
| **Atrophy of putamen^1)^** | 25/29 | - | - |
| **SBR Right** | 1.54 ± 0.65 | - | - |
| **Left** | 1.39 ± 0.60 | - | - |
| **NRC_SN_ Right** | 16.16 ± 9.04 | 31.89 ± 9.82 | 0.000* |
| **Left** | 21.39 ± 10.25 | 39.82 ± 9.73 | 0.000* |
| **NRC_LC_ Right** | 1.56 ± 1.10 | 2.40 ± 1.19 | 0.013* |
| **Left** | 2.54 ± 1.18 | 3.41 ± 1.17 | 0.012* |

Data on continuous variables are expressed as the mean ± SD. The *P* value represents the result of a comparison between the control and MSA-P groups. * *P* < 0.05 indicates statistical significance. ^1)^ Number of patients with atrophy in the putamen at MRI and SPECT examinations. ^2)^ To obtain age and sex matched controls, 22 patients with essential tremor who underwent 3-Tesla neuromelanin-sensitive MRI were enrolled. Abbreviations: MSA-P, multiple system atrophy with parkinsonism; NRC_LC_, neuromelanin-related contrast in the locus coeruleus; NRC_SN_, neuromelanin-related contrast in the substantia nigra; SBR, specific binding ratio.

**Supplemental Table 3. Linear regression between the NRC_SN_ and SBR in the early to intermediate stage of DLB, PD, PSP, CBS, and MSA-P**

|  | DLB  n = 29 | PD  n = 52 | PSP  n = 31 | CBS  n = 30 | MSA-P  n = 29 |
| --- | --- | --- | --- | --- | --- |
| SBR Right  Left | 0.99 ± 0.65  1.01 ± 0.68 | 1.63 ± 0.72  1.58 ± 0.70 | 1.78 ± 0.89  1.76 ± 0.87 | 2.17 ± 1.02  1.93 ± 1.06 | 1.54 ± 0.65  1.39 ± 0.60 |
| NRC_SN_ Right  Left | 8.86 ± 4.73  14.57 ± 7.90 | 12.72 ± 6.70  17.56 ± 7.74 | 12.71 ± 6.56  17.01 ± 6.81 | 15.26 ± 9.50  22.59 ± 15.89 | 16.16 ± 9.04  21.39 ± 10.25 |
| SBR-based  MA side | *R*^2^ = 0.144  *P* = 0.042* | *R*^2^ = 0.165  *P* = 0.003** | *R*^2^ = 0.069  *P* = 0.155 | *R*^2^ = 0.204  *P* = 0.012** | *R*^2^ = 0.026  *P* = 0.403 |
| NRC_SN_-based  MA side | *R*^2^= 0.059  *P* = 0.204 | *R*^2^ = 0.147  *P* = 0.005** | *R*^2^ = 0.038  *P* = 0.295 | *R*^2^ = 0.214  *P* = 0.010** | *R*^2^ = 0.108  *P* = 0.082 |
| (SBR + NRC_SN_)-based MA side | *R*^2^= 0.109  *P* = 0.080 | *R*^2^ = 0.235  *P* = 0.000** | *R*^2^ = 0.071  *P* = 0.149 | *R*^2^ = 0.221  *P* = 0.009** | *R*^2^ = 0.029  *P* = 0.376 |
| Clinically-defined MA side | - | *R*^2^ = 0.228  *P* = 0.001**  (n = 49) | *R*^2^ = 0.054  *P* = 0.405  (n = 15)^1)^ | *R*^2^ = 0.229  *P* = 0.007**  (n = 30) | *R*^2^ = 0.039  *P* = 0.393  (n = 21) |
| Laterality of parkinsonism^2)^ | 15/29^3)^  51.7% | 49/52  94.2% | 17/31^4)^  54.8% | 30/30  100.0% | 21/29  72.4% |

Data on continuous variables are expressed as the mean ± SD. After adjusting for the interhemispheric asymmetry of neuromelanin-related MRI contrast by using the Z-score, age-adjusted linear regression between the NRC_SN_ and SBR was performed in the most affected side of the hemispheres for the various definitions. Results for the DLB and PD groups were cited in a previous study [1]. *Significance was set at *P* <0.05. **Significant result following Bonferroni FDR correction. ^1)^PSP-RS with laterality of parkinsonism. ^2)^Laterality of parkinsonism was defined by at least two of the motor manifestations of parkinsonism (bradykinesia/akinesia, tremor at rest, and muscular rigidity) showing the laterality on the ipsilateral side. ^3)^Seven patients with DLB had no parkinsonism. ^4)^Three patients with PSP had no parkinsonism. Abbreviations: CBS, corticobasal syndrome; DLB, dementia with Lewy bodies; LA, least affected; MA, most affected; MSA-P, multiple system atrophy with predominant parkinsonism; NRC_SN_, neuromelanin-related contrast in the substantia nigra; PD, Parkinson’s disease; PSP, progressive supranuclear palsy; PSP-RS, PSP-Richardson's syndrome; *R*^2^, coefficient of determination; SBR, specific binding ratio.

Ref. 1. M. Okitsu, K. Sugaya, Y. Nakata, T. Kawazoe, J. Ikezawa, R. Okiyama, et al. Degeneration of nigrostriatal dopaminergic neurons in the early to intermediate stage of dementia with Lewy bodies and Parkinson's disease. J Neurol Sci 449 (2023) 120660.


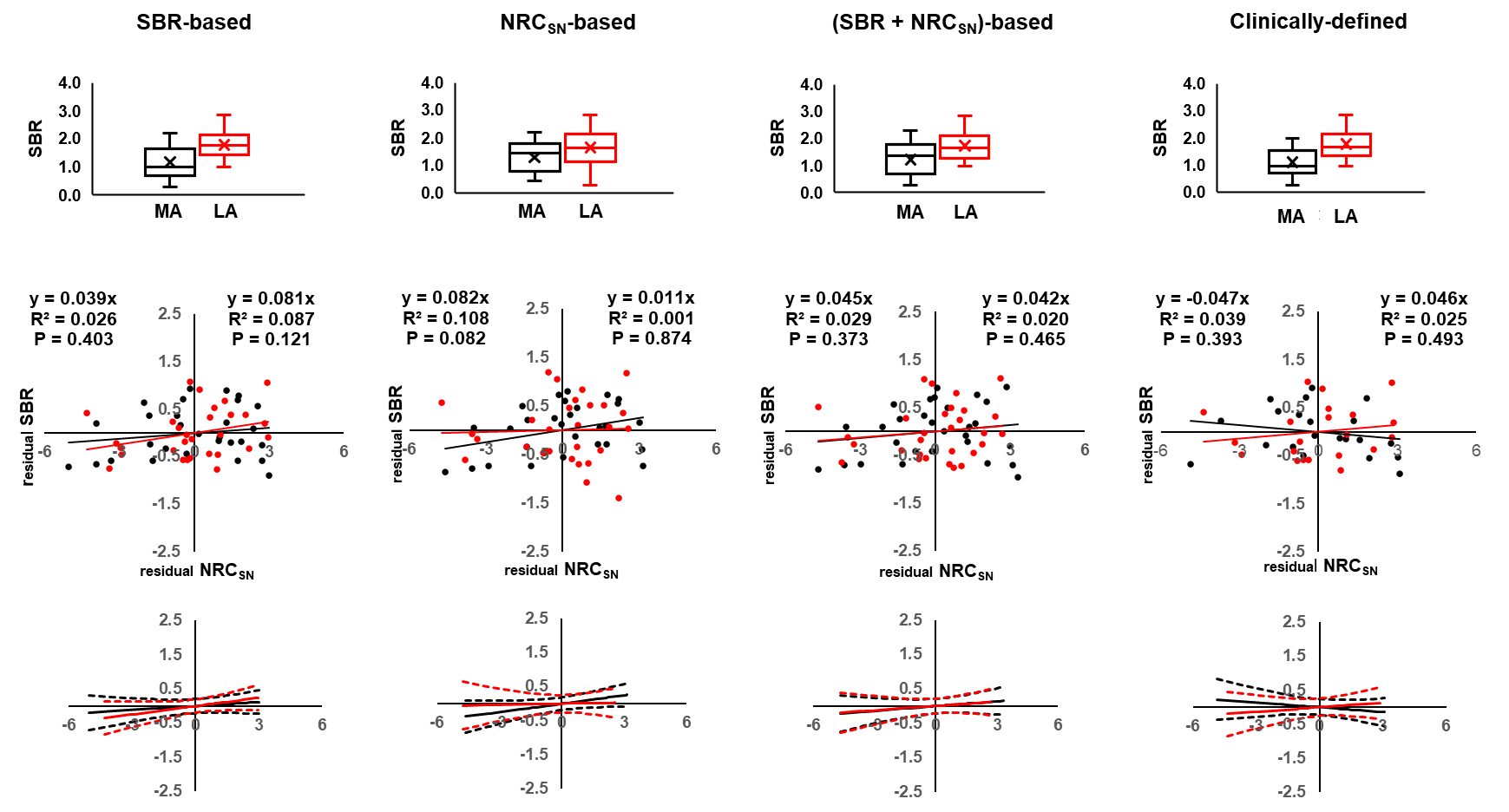


**Supplemental Figure 2.**

**Linear regression analysis of the corrected NRC_SN_ and SBR on the most and least affected (MA and LA) sides in MSA-P**

Linear regression analysis of the corrected NRC_SN_ and SBR on the SBR-based MA (black) and LA (red) sides, NRC_SN_-based MA and LA sides, on the (SBR + NRC_SN_)-based MA and LA sides, and clinically defined MA and LA sides was performed using the Z-score of the In(NRC_SN_).

Upper portion: Box plots of the SBR distribution comparing the SBR values. The line in the box represents the median value, and x represents the mean value.

Middle portion: Results of linear regression analysis of the corrected NRC_SN_ (residual NRC_SN_) and SBR (residual SBR) after adjusting for individual differences in age.

Lower portion: 95% confidence interval for each linear model (broken lines).

Abbreviations: MA, most affected; LA, least affected; SBR, specific binding ratio; NRC_SN_, neuromelanin-related contrast in the substantia nigra; MSA-P, parkinsonism-predominant multiple system atrophy.
